# Binding Multilateral Framework for South Asian Air Pollution Control: An Urgent Call for SAARC-UN Cooperation

**DOI:** 10.3390/ijerph22111628

**Published:** 2025-10-26

**Authors:** Shyamkumar Sriram, Saroj Adhikari

**Affiliations:** Department of Rehabilitation and Health Services, University of North Texas, Denton, TX 76203, USA; shyamkumar.sriram@unt.edu

**Keywords:** PM_2.5_ mitigation, trans-sectoral governance, South Asia, transboundary air pollution, regional cooperation

## Abstract

South Asia’s worsening air pollution crisis represents one of the most urgent public health and environmental challenges of the 21st century. Nearly two billion people—over one-quarter of the global population—reside in this region, where air quality levels routinely exceed World Health Organization (WHO) guidelines by factors of 10 to 15. This has translated into an unprecedented health burden, with approximately two million premature deaths annually, widespread chronic respiratory and cardiovascular disease, and rising economic losses. According to recent World Bank estimates, welfare losses amount to over 5% of regional GDP, a figure far exceeding the projected costs of coordinated mitigation. Despite this, South Asia continues to lack a binding regional framework capable of addressing its shared airshed. Existing cooperative efforts—such as the Malé Declaration on Control and Prevention of Air Pollution (1998)—have provided a useful platform for dialog and pilot monitoring, but they remain voluntary, under-resourced, and insufficient to manage the transboundary nature of the crisis. National-level programs, including India’s National Clean Air Programme (NCAP), Bangladesh’s National Air Quality Management Plan (NAQMP), and Nepal’s National Air Quality Management Action Plan (AQMAP), demonstrate domestic commitment but are constrained by fragmentation, limited financing, and lack of regional integration. This gap represents the central knowledge and governance challenge that prompted the present commentary. To address it, we propose a dual-track architecture designed to institutionalize binding regional cooperation. Track A would establish a United Nations-anchored South Asian Transboundary Air Pollution Protocol, under the auspices of the United Nations Environment Programme, the World Health Organization (WHO), and the United Nations Economic and Social Commission for Asia and the Pacific (UNESCAP). This protocol would codify legally enforceable emission standards, compliance committees, financial mechanisms, and harmonized monitoring. Track B would establish a South Asian Association for Regional Cooperation (SAARC) Prime Ministers’ Council on Air Quality (SPMCAQ) to provide political leadership, align domestic implementation, and authorize rapid responses to cross-border haze events. Lessons from the Indian Ocean Experiment, the ASEAN Agreement on Transboundary Haze Pollution, and Europe’s Convention on Long-Range Transboundary Air Pollution demonstrate that legally binding agreements combined with high-level political ownership can achieve durable reductions in pollution despite geopolitical tensions. By situating South Asia within these global precedents, the proposed framework provides a pragmatic, enforceable, and politically resilient pathway to protect health, reduce economic losses, and deliver cleaner air for nearly one-quarter of humanity.

## 1. The Urgent Case for Binding Action

### 1.1. Health Crisis at Unprecedented Scale

South Asia’s air pollution burden is unparalleled in its combination of scale, intensity, and persistence. In 2021, 37 of the world’s 40 most polluted cities were in South Asia [1], reflecting sustained exceedances of WHO guideline values across multiple pollutants, particularly PM 2.5. In urban centers such as Delhi, Dhaka, and Kathmandu, seasonal inversions, biomass burning, industrial emissions, and transport sources combine to drive PM 2.5 concentrations exceeding the World Health Organization’s (WHO) recommended guideline of 5 μg/m^3^ by up to 15 times [2], yielding a chronic exposure environment for large populations. This exposure profile has translated into the world’s highest burden of pollution-related respiratory and cardiovascular disease, with mounting evidence of causal links to diabetes, neurocognitive decline, adverse pregnancy outcomes, and child developmental deficits. As a conservative estimate, ~2 million premature deaths annually across the region are attributable to fine particulate exposure—losses that concentrate among the poorest, the very young, and the elderly, thereby amplifying health inequities [3].

The burden is not limited to mortality. Morbidity—manifested as chronic obstructive pulmonary disease, ischemic heart disease, stroke, asthma exacerbations, and acute lower respiratory infections [4,5]—imposes long-term disability, diminished labor productivity, and increased out-of-pocket health expenditures [6,7]. Children under five face stunted lung development and heightened lifetime risk of respiratory illness [8], while older adults and workers in informal sectors experience cumulative exposure without social protection. Health systems, already constrained in many parts of the region, endure recurring spikes in admissions during haze episodes [9], stretching capacity and crowding out care for other conditions. Against this backdrop, incremental or purely local interventions are structurally inadequate: the crisis is regional by nature and therefore requires a legally coordinated regional response.

### 1.2. Economic Imperative: Healthcare Costs Exceed Mitigation Investment

The economic and health costs of inaction far exceed the investments required for coordinated mitigation. According to World Bank, 2021 report, welfare losses related to air pollution in South Asia are over 7% of GDP, reflecting mortality, morbidity, and productivity effects. Country-level estimates such as Bangladesh’s ~7% of GDP, India’s 1.4% and Pakistan’s 6% highlight the magnitude of national impacts. These losses cascade through household budgets (via out-of-pocket spending), firm-level output (via absenteeism and diminished cognitive/physical performance), and public finances (via healthcare and social protection) [2]. Disability-adjusted life years (DALYs) lost to pollution-related illnesses translate into structural drag on growth, compromise human capital accumulation, and exacerbate intergenerational poverty traps [10].

By contrast, the incremental annual cost of comprehensive clean-air measures for South Asia is estimated at US$ 5.7 billion per year, which is lesser than one percent of regional GDP through 2030 [11]. When benchmarked against current losses, this implies a very high social return even under conservative assumptions. The mitigation benefit–cost ratio remains strongly favorable. In short, the region is paying far more to tolerate pollution than it would to abate it. This scenario demands for binding policy that internalizes cross-border externalities and coordinates action at scale.

### 1.3. Transboundary Nature Demands Binding Cooperation

South Asia functions as a shared airshed, where emissions originating in one jurisdiction routinely affect downwind populations in another. Foundational work—from the 1999 Indian Ocean Experiment (INDOEX) campaign to subsequent analyses of the Atmospheric Brown Cloud—documented thick layers of anthropogenic aerosols over the northern Indian Ocean with substantial contributions from South Asia [12]. Chemical transport modeling and long-range transport studies now quantify cross-border contributions to PM 2.5, with winter haze episodes often producing synchronous regional deterioration in air quality [3]. Seasonal meteorology, including synoptic wind patterns and boundary-layer dynamics, regularly moves pollutants across national borders throughout the Indo-Gangetic Plain and into the Himalayan foothills, including the Hindu Kush Himalaya region [13].

These dynamics render purely national strategies insufficient. Even ambitious domestic controls will be diluted by inflows from neighbors unless harmonized standards, joint monitoring, and coordinated enforcement are implemented across borders. The implication is straightforward: binding multilateral cooperation is not merely desirable; it is a necessary condition for achieving sustained and equitable exposure reductions across the region.

Despite decades of concern, regional agreements in South Asia have been limited in scope and effectiveness. The Malé Declaration on Control and Prevention of Air Pollution (1998) provided an important early platform for cooperation but lacked binding commitments, time-bound targets, and enforcement mechanisms [14]. National programs—such as India’s National Clean Air Programme (NCAP) [15] with its PRANA (Portal for Regulation of Air Pollution in Non-Attainment Cities) platform, Bangladesh’s National Air Quality Management Plan (2024–2030) [16], and Nepal’s National Air Quality Management Action Plan (2021–2025) [17]—demonstrate domestic progress but remain constrained by fragmented standards, insufficient financing, and weak cross-border coordination [18]. By comparison, the ASEAN Agreement on Transboundary Haze Pollution (2002) illustrates the benefits of binding obligations, joint monitoring, and enforcement, though challenges of compliance persist [19]. Taken together, these experiences show that voluntary or purely national approaches are inadequate. This gap provides the rationale for a legally binding, regionally coordinated framework tailored to South Asia’s shared airshed.

### 1.4. Current Policy Landscape and Gaps

Despite numerous efforts to address air pollution, progress in South Asia remains limited. Existing policies are fragmented across environment, health, transport, energy, and agriculture sectors, resulting in misaligned standards, overlapping mandates, and weak cross-sectoral accountability. Regulatory bodies often face capacity constraints, with insufficient monitoring coverage, limited laboratory resources for source apportionment, and inadequate inspection bandwidth—leading to weak enforcement of emission controls and land-use regulations. Financing for clean air is typically short-term and projectized, leaving local governments without predictable resources to sustain implementation [20].

Regional cooperation mechanisms exist but remain largely non-binding and under-resourced. Voluntary frameworks such as the Malé Declaration have supported dialog and pilot monitoring, yet they lack enforceable commitments, time-bound targets, and credible compliance procedures. National initiatives—including India’s NCAP with its PRANA portal and Bangladesh’s NAQMP (2024–2030)—generate important domestic momentum, but their regional impact depends on harmonized standards, synchronized timelines, and interoperable data protocols. Overall, policy intent consistently outpaces institutional capability, and voluntary arrangements fail to address cross-border externalities effectively.

Existing multilateral and international frameworks provide a critical foundation for a binding regional mechanism. The WHO Global Air Quality Guidelines (2021) offer health-based reference values for PM 2.5, PM_10_, NO_2_, SO_2_, and O_3_, which can anchor regional targets and guide national reduction pathways. The Malé Declaration illustrates the potential for regional cooperation on transboundary air pollution monitoring and dialog, serving as a platform that could be strengthened into a binding mechanism. International legal precedents, including the Trail Smelter Arbitration’s “no-harm” principle and the state responsibility obligations codified in the Stockholm (1972) and Rio (1992) Declarations, provide normative grounding for cross-border mitigation responsibilities. National statutes and programs—such as India’s Air (Prevention & Control) Act (1981) and NCAP, and Bangladesh’s NAQMP—demonstrate domestic scaffolding that could be aligned with regional standards, timelines, and monitoring protocols.

Building on these instruments, a PMO-anchored, trans-sectoral framework could establish enforceable coordination, compliance, and financing mechanisms. By integrating health-based benchmarks, legal obligations, harmonized monitoring, and national programs, such a framework would address the limitations of fragmented policies and voluntary arrangements, providing the foundation for effective regional action against air pollution.

Table 1 maps existing policy instruments, highlighting how each can contribute to a binding, coordinated, and enforceable regional air quality strategy under PMO leadership.

## 2. Proposed Binding Framework: Dual-Track Architecture

This proposal adopts a dual-track architecture to manage South Asia’s airshed: a UN-anchored binding protocol (Track A) to set and enforce legal obligations, and a SAARC Prime Ministers’ Council on Air Quality (Track B) to provide head-of-government leadership, align budgets, and sustain cooperation. National PMO Air Coordination Units (PACUs) translate regional commitments into domestic action. The framework is explicitly aligned with the World Bank’s roadmap and is built to operationalize it: Phase I (monitoring, science, institutions) is delivered via standardized MRV, a Technical Secretariat, and a Scientific Advisory Panel; Phase II (cost-effective sectoral interventions) is driven by PACUs with SPMCAQ oversight; Phase III (economic instruments and financing) is anchored by binding standards, market tools, and a regional fund with results-based verification.

### 2.1. Track A: UN-Anchored Binding International Protocol

Legal foundation: Drawing on the Trail Smelter line of reasoning and Principle 21 (Stockholm)/Principle 2 (Rio), a South Asian Transboundary Air Pollution Protocol under UN auspices like the World Health Organization (WHO), United Nations Environment Programme (UNEP) and the United Nations Economic and Social Commission for Asia and the Pacific (UNESCAP) would establish legally binding obligations for participating states. The protocol’s design emphasizes technical neutrality, predictable compliance, and transparent reporting.

Core provisions (proposed):

- PM 2.5 exposure reduction trajectory: An indicative 50% reduction by 2035 (relative to an agreed baseline), aligned with WHO guidelines and interim targets, to be country-differentiated via nationally determined implementation pathways and periodic scientific review.
- Sectoral emission standards: Harmonized, legally binding limits for power, industry (including brick kilns), transport, waste burning, agriculture/residue burning, and household energy, with time-bound adoption and phase-out schedules for high-emitting technologies.
- Cross-border EIA (Environmental Impact Assessment): Mandatory prior notification and assessment for projects with likely transboundary air quality impacts, including safeguards for vulnerable communities.
- Data infrastructure: Standardized monitoring protocols, calibration and QA/QC, open data sharing, and joint receptor/source apportionment studies to inform equitable burden-sharing.
- Compliance and remedies: A Compliance Committee empowered to assess non-compliance, recommend corrective action, and trigger financial consequences that recycle resources to a regional mitigation fund.

Institutional structure:

- Technical Secretariat: (hosted by UNEP, with WHO and UNESCAP participation) to manage reporting, develop technical guidance, and support capacity building.
- Compliance Committee: an independent body to review national reports, conduct expert assessments, and oversee graduated responses to non-compliance.
- Scientific Advisory Panel: to update exposure-response functions, source apportionment methods, and technology roadmaps, ensuring evidence-based target-setting.
- South Asia Air Quality Fund: to provide predictable financing for abatement, monitoring, and health adaptation, combining mandatory contributions and donor co-financing.

### 2.2. Track B: SAARC Prime Ministers’ Council on Air Quality (SPMCAQ)

Political rationale: Durable progress requires head-of-government leadership capable of mandating inter-ministerial action, aligning budgets, and sustaining cooperation during political cycles and bilateral frictions. A PM-level body signals national priority and provides the diplomatic bandwidth to overcome coordination failures.

Design features:

- Membership: PMO-designated focal points (Director/Secretary level) from each SAARC member state.
- Rotating chair: Six-month rotation to mitigate dominance concerns and encourage shared ownership.
- Secretariat: A dedicated three-person desk at the SAARC Secretariat to convene meetings, track actions, and serve as the interface with Track A institutions.
- Technical partnership: Direct liaison with the UN-anchored Secretariat, enabling two-way translation between legal obligations and operational delivery.

Operational mandate:

- National implementation oversight of protocol obligations with quarterly PMO-level reviews.
- Cross-border emergency response authorization within a defined window (e.g., 48 h) for forest fires, industrial accidents, or extreme haze—the precise window to be finalized during negotiations for feasibility and fairness.
- Financing gateway: Endorse country proposals to the regional fund, prioritize high-impact, equity-centered investments, and de-risk donor participation.
- Dispute mitigation: Mediate emerging bilateral issues prior to formal compliance procedures, maintaining cooperation continuity.

### 2.3. National Implementation: PMO Air Coordination Units (PACU)

To translate regional obligations into domestic action, each country would establish a PACU under the Office of the Prime Minister with authority to perform the following:

- To chair inter-ministerial technical committees (environment, energy, transport, agriculture, health, finance, industry, social welfare).
- To develop a National Implementation Roadmap with binding timelines, responsible agencies, and budget tags.
- To coordinate emergency measures during transboundary incidents (e.g., short-term emission curbs, traffic management, targeted advisories for vulnerable groups).
- To act as national focal point for regional fund access, ensuring proposals meet technical criteria, equity screens, and co-benefit accounting.
- To institutionalize accountability via public dashboards, performance contracts for agencies, and annual parliamentary reporting.

This PMO centering addresses fragmentation by creating a single locus of authority that can reconcile sectoral trade-offs (e.g., power security vs. emissions), sequence reforms, and sustain implementation through political turnover.

**National-Level Implementation Examples**

To ensure feasibility, the proposed protocols can be operationalized through phased national implementation roadmaps:

- India (NCAP/PRANA): Integration of national city-level targets with binding regional standards; milestone: 25% reduction in PM 2.5 in non-attainment cities by 2030.
- Bangladesh (NAQMP 2024–2030): Alignment of action plans with regional emission ceilings; milestone: phase-out of uncontrolled brick kilns by 2027.
- Nepal (AQMAP 2021–2025): Expansion of monitoring networks to be fully interoperable with regional data platforms by 2025.
- Pakistan (Punjab Clean Air Plan): Adoption of harmonized vehicular emission standards by 2028.

Each national Prime Minister’s Office (PMO)-led Air Coordination Unit would be responsible for translating regional commitments into domestic law, with 5-year review cycles and interim milestones monitored by a joint compliance committee.

## 3. Economic and Financial Architecture

A robust economic and financial architecture is essential to sustain the dual-track framework and ensure effective implementation of air quality action plans. The proposed approach emphasizes national ownership, whereby each participating country legally commits to allocate a defined portion of its domestic fiscal budget to implement its national action plan. This mechanism secures predictable funding without creating disputes over a pooled regional fund, while ensuring alignment between national priorities and regional air quality objectives.

Complementing domestic commitments, conditional donor financing provides an additional layer of support. Donors—including multilateral development banks, the Green Climate Fund, and bilateral partners—would release funds only after verification that the government has allocated its committed budget. This approach strengthens accountability, incentivizes timely allocation of resources, and reinforces the credibility of national implementation. Where feasible, countries may also leverage innovative domestic financing mechanisms, such as Paris-aligned carbon market linkages, health impact bonds, or infrastructure bonds for clean energy and transport, to further accelerate abatement measures. Together, this architecture combines legal fiscal commitment with targeted external support, creating a sustainable, incentive-compatible financial framework for airshed-wide air quality management in South Asia.

## 4. Addressing Regional Political Dynamics: Conflict-Resilience Design

The proposed dual-track architecture is deliberately structured to withstand political volatility in South Asia, where bilateral disputes often impede sustained cooperation. By entrusting the administration of the binding legal protocol to a neutral UN mechanism, the framework safeguards technical continuity and enforcement even when regional diplomacy is strained. In parallel, the SAARC council, anchored within the Prime Minister’s Offices, ensures political ownership, peer accountability, and legitimacy at the highest executive level. This layered arrangement allows technical processes to remain insulated from diplomatic turbulence, while political leadership retains a central role in guiding strategic direction. Pre-agreed emergency protocols further guarantee that essential public-health measures—such as responses to large-scale fires, cross-border haze episodes, or industrial accidents—remain operational during periods of heightened tension. Additionally, financial incentives, particularly conditional access to regional or multilateral funds, foster positive-sum dynamics by rewarding compliance and sustained engagement. In doing so, the framework pragmatically balances national sovereignty sensitivities with the collective action imperatives required for effective airshed-wide management.

**Lessons from Regional Case Studies and Political Dynamics**

Historical precedents demonstrate the potential benefits of binding regional frameworks. The ASEAN Agreement on Transboundary Haze Pollution (2002) was the world’s first legally binding regional environmental agreement of its kind. By mandating regional monitoring, coordinated emergency responses, and compliance reviews, it reduced the severity of haze episodes despite persistent challenges in Indonesia’s enforcement capacity [19]. Similarly, the UNECE Convention on Long-Range Transboundary Air Pollution (CLRTAP) in Europe has achieved significant reductions in sulfur dioxide and nitrogen oxides, showing how binding commitments and joint monitoring can sustain cooperation even amid political tensions [33]. Comparable initiatives in East Asia, such as the Tripartite Environment Ministers Meeting among China, Japan, and South Korea, have demonstrated the feasibility of regional emission reduction through cooperative monitoring [34], though their voluntary nature highlights the importance of enforceable mechanisms in the South Asian context.

In South Asia, political dynamics such as long-standing disputes between India and Pakistan, or asymmetrical capacities between smaller Himalayan states and larger economies, could hinder implementation. The proposed framework mitigates these risks by (a) entrusting enforcement to a neutral UN-anchored compliance body, (b) embedding dispute mediation within the SAARC Prime Ministers’ Council on Air Quality, and (c) conditioning access to regional funds on compliance with obligations. These design features insulate technical implementation from political volatility, while financial incentives foster cooperative rather than adversarial dynamics.

## 5. Theory of Change Framework for Regional Airshed-Wide Air Quality Management

This theory of change illustrates how the proposed dual-track framework (multilateral cooperation + binding agreements) can operationalize the World Bank’s roadmap for air quality management in South Asia. It provides a logical pathway from inputs to long-term impacts, while leaving space for each nation to design its own monitoring and evaluation system (Table 2).

## 6. Conclusions: The Imperative for Immediate Action

Historical precedents from Europe, Southeast Asia, and the Rhine basin demonstrate that binding regional agreements, credible MRV systems, and sustained financing can overcome political tensions and deliver lasting environmental gains. The UNECE CLRTAP [35], the ASEAN Haze Agreement [36], and the Rhine Action Program [37] all show that legal enforceability and shared monitoring enable durable cooperation even amid geopolitical strain. These lessons are directly transferable to South Asia, where air pollution is both transboundary and deeply interlinked across airsheds. Adopting a similar framework would not only institutionalize cooperation but also unlock enormous economic and health dividends, with evidence indicating that every dollar invested could yield returns exceeding eighteen dollars. In this light, delaying coordinated action is far costlier than the investments required, and a legally binding, regionally coordinated approach offers the most viable pathway to clean air and healthier futures in South Asia.

## Figures and Tables

**Table 1:** Existing International, Regional, and National Frameworks Relevant to Air Pollution Control in South Asia.

Instrument/Policy	Scope and Relevance	Source
WHO Global Air Quality Guidelines (2021)	International health-based guideline values for PM 2.5, PM_10_, NO_2_, SO_2_, O_3_. Provides evidence-based benchmarks for setting national and regional targets.	[21]
Malé Declaration on Control & Prevention of Air Pollution (1998, SACEP)	Voluntary regional declaration among South Asian countries to foster cooperation in monitoring and managing transboundary air pollution. Non-binding.	[22]
Trail Smelter Arbitration/No-Harm Principle	International law precedent affirming state responsibility to prevent transboundary environmental harm.	[23]
Stockholm Declaration (1972)/Rio Declaration (1992)	Global declarations establishing state responsibility to avoid cross-border environmental damage and cooperate in environmental protection.	[24]
India—Air (Prevention & Control) Act (1981); National Clean Air Programme (2019)	Statutory framework for air pollution control, city-specific targets, and PRANA monitoring portal.	[25]
Bangladesh—National Air Quality Management Plan (2024–2030)	National strategy for comprehensive air quality management with time-bound interventions.	[26]
Pakistan—National Environmental Quality Standards (NEQS, revised 2010); Punjab Clean Air Action Plan (2019)	Legally enforceable standards for industrial and vehicular emissions; provincial plan addressing smog and transboundary haze.	[27]
Nepal—Environment Protection Act (2019) and National Air Quality Management Action Plan (2021–2025)	Provides legal mandate and strategic plan for emission reduction, urban air quality management, and monitoring networks.	[28]
Bhutan—Environmental Protection Act (2007); Bhutan Air Quality Standards (2010)	Establishes national standards for ambient air quality and mandates pollution monitoring.	[29]
Sri Lanka—National Environmental Act (1980, amended); Vehicle Emission Testing Program (2008)	Legal framework for pollution control and regulatory programs targeting vehicular emissions.	[30]
Afghanistan—National Environmental Protection Act (2007)	Legal mandate for pollution control, though implementation capacity remains limited due to conflict and institutional weakness.	[31]
ASEAN Agreement on Transboundary Haze Pollution (2002)	While outside SAARC, provides a transferable model for binding regional cooperation on air pollution that can inform South Asian frameworks.	[32]

**Table 2:** Theory of Change Framework for Regional Air Quality Management.

Level	Description
Inputs	- Political commitment at national and regional levels; - Regional cooperation platforms (SAARC, BIMSTEC, UN agencies); - Financial and technical resources from governments, donors, and multilateral banks; - Scientific expertise, data infrastructure, and technological tools (sensors, modeling, AI/ML applications); - Engagement of civil society, private sector, and academia.
Processes/Activities	- Establish regional emissions inventories and data-sharing systems; - Harmonize sectoral standards (transport, energy, industry, agriculture, waste); - Develop cross-border protocols for airshed management; - Build institutional and scientific capacity at national and sub-national levels; - Promote behavior-change campaigns for households, farmers, and small enterprises; - Facilitate pilot projects on clean technologies and fuels.
Outputs	- Functional regional knowledge hub and open-data platform; - Adopted guidelines for coordinated airshed management; - National and regional policies aligned with shared standards; - Increased adoption of clean technologies in targeted sectors; - Documented cross-border cooperation cases (joint programs, enforcement, or financing mechanisms).
Outcomes (Medium-term)	- Reduced emissions from key polluting sectors (transport, brick kilns, agriculture burning, household energy); - Increased compliance with harmonized standards across the airshed; - Improved institutional collaboration across borders and sectors; - Enhanced public awareness and participation in clean air initiatives; - Demonstrable progress toward WHO Interim Target 1 (≤35 µg/m^3^).
Impacts (Long-term)	- Sustainable reduction of PM_2.5_ and other air pollutants to levels aligned with WHO guidelines; - Improved population health (fewer premature deaths and illnesses); - Strengthened regional solidarity and cooperative governance; - Economic benefits from reduced healthcare costs, increased productivity, and cleaner ecosystems; - Contribution to climate co-benefits (lower black carbon and GHG emissions).

## Data Availability

The original contributions presented in this study are included in the article. Further inquiries can be directed to the corresponding author.
